# The structural stability of negative symptoms over time

**DOI:** 10.1192/bjo.2023.600

**Published:** 2023-11-03

**Authors:** Noham Wolpe, Emilio Fernandez-Egea

**Affiliations:** Department of Physical Therapy, The Stanley Steyer School of Health Professions, Faculty of Medicine, Tel Aviv University, Tel Aviv, Israel; Sagol School of Neuroscience, Tel Aviv University, Tel Aviv, Israel; and Department of Psychiatry, University of Cambridge, Cambridge, UK; Department of Psychiatry, University of Cambridge, Cambridge, UK; and Cambridge Psychosis Centre, Cambridgeshire and Peterborough NHS Foundation Trust, Cambridge, UK

**Keywords:** Negative symptoms, motivation, network analysis, psychotic disorders/schizophrenia, longitudinal analysis

## Abstract

Negative symptoms remain poorly understood and treated despite their huge impact on patients’ lives and clinical outcomes. This is partly because of ongoing debates about the clinical constructs underlying negative symptoms. A longitudinal analysis of the structure of negative symptoms presented in *BJPsych Open* reports striking temporal stability of symptom structure, which behaves as a few independent domains. This further underscores the need to address specific symptom domains when considering interventions or pathophysiology studies.

In both DSM-5-TR and ICD-11, negative symptoms are clustered into a single criterion within the clinical diagnostic criteria for schizophrenia. This is despite the growing evidence suggesting that negative symptoms are a few distinct symptoms that behave differently over time and have different underlying pathophysiology.^1^ Moreover, an ongoing debate exists about what clinical constructs underlie these symptoms. For example, in DSM-5-TR, criterion A5 reads: ‘Negative symptoms (i.e., diminished emotional expression or avolition)’. By contrast, in ICD-11, the equivalent criterion (criterion e) reads: ‘Negative symptoms such as affective flattening, alogia or paucity of speech, avolition, asociality and anhedonia’. The erroneous clustering of negative symptoms into one clinical construct and the disagreements about those underlying constructs hamper the development of new treatments for these debilitating symptoms.^2^

For the past three decades, research studies have attempted to investigate the underlying statistical structure of negative symptoms in the hope that these analyses will inform our understanding of the clinical constructs underlying negative symptoms. Results have been inconsistent, revealing a two-, three-, four- or five-factor structure to negative symptoms, typically measured using clinician-rated scales.^3,4^ In 2005, a National Institute of Mental Health (NIMH) ‘consensus development conference’ was held, leading to a consensus statement regarding the five clinical constructs constituting negative symptoms: anhedonia, asociality, avolition, blunted affect and alogia. This led to the development of ‘second-generation’ scales of negative symptoms which cover these five consensus ‘domains’, but the discussion as to whether all five domains are clinically separable in a meaningful way is yet to be resolved.

## Rucci et al's study

### Longitudinal network analyses

In a recent issue of *BJPsych Open*, Rucci et al use longitudinal network analyses to examine the structure of negative symptoms and their stability over time.^5^ In an impressive sample size of 612 community-dwelling individuals with schizophrenia (from the prolific Italian Network for Research on Psychoses), they found a striking temporal stability of negative symptom structure after 4-year follow-up compared with baseline. Although some changes were observed in global strength invariance test scores, suggesting more minor associations between symptoms at follow-up relative to baseline, a community analysis revealed a similar symptom structure, with four (at baseline) and five (at follow-up) communities best explaining the data. The study supports the more complex structure for negative symptoms, with four or five symptom domains remaining largely stable over time.

This study by Rucci et al elegantly illustrates the added value of more advanced statistical methods, such as longitudinal network analyses, in a relatively large cohort of patients. Further strengths of this study include using a second-generation assessment tool for negative symptoms, which is yet to be the standard in this line of research. Lastly, the relatively long follow-up period of 4 years adds significant merit to this research and further underscores the stability of symptoms over time.

### Symptom domains and temporal stability

The study makes at least two significant contributions to the literature on negative symptoms. First, it adds to our understanding of the constructs or domains underlying negative symptoms, supporting the more complex five-factor models. However, it remains to be seen whether, and if so, how the debate about these domains can be resolved. In addition to studies like Rucci et al's, further questions should be addressed if we are ever to resolve the two- versus five-factor conundrum. One critical question which needs to be addressed is the clinical significance of using more complex models of negative symptoms. Does using a more complex five-factor model of negative symptoms inform us clinically, for example does it improve the prediction of clinical outcome and response to future treatment? Similarly, is using the more complex models advantageous because they give us better biological insights about symptom mechanisms? The jury is still out. Another critical question surrounds the importance of individual differences. As these methods largely involve pooling patient data to discover the mean symptom structure, it is possible that the inconsistent results on symptom structure can be explained by patient heterogeneity. Although some individuals with schizophrenia may express five independent domains of negative symptoms, others, perhaps with more chronic illness, may express the less-complex two independent domains. To answer this, significantly larger sample sizes will be required.

For the second contribution, although Rucci et al may not resolve the debate on the number of underlying domains of negative symptoms, their study strongly supports the temporal stability of these symptoms. An open question for future research concerns the effect of other clinical variables on symptom structure: variability in secondary causes of negative symptoms, such as psychosis and depression, may influence the relationship between symptoms over time, influencing structure stability. For example, drug-induced sedation specifically influences motivation and pleasure but does not affect emotional expressivity.^6^

The overall stability of negative symptom domains over time suggests that future clinical trials should target specific domain(s), rather than negative symptoms as a whole. This may explain the failure of previous clinical trials in improving negative symptoms. This study and similar findings on the multi-faceted nature of negative symptoms suggest that now is the time to reignite the interest of pharmaceutical companies in negative symptom treatment. Such clinical trials are needed to improve our treatment of these debilitating symptoms finally.

## Data Availability

Data availability is not applicable to this article as no new data were created or analysed in this study.

## References

[1] Galderisi S, Mucci A, Buchanan RW, Arango C. Negative symptoms of schizophrenia: new developments and unanswered research questions. Lancet Psychiatry 2018; 5: 664–77. 29602739 10.1016/S2215-0366(18)30050-6

[2] Fernandez-Egea E, Wolpe N. Is it time to change the term negative symptoms? Eur Neuropsychopharmacol 2022; 59: 7–8. 10.1016/j.euroneuro.2022.03.004 35378383

[3] Strauss GP, Esfahlani FZ, Galderisi S, Mucci A, Rossi A, Bucci P, et al. Network analysis reveals the latent structure of negative symptoms in schizophrenia. Schizophr Bull 2019; 45: 1033–41. 30256991 10.1093/schbul/sby133 PMC6737465

[4] Li S, Liu C, Zhang J, Wang L, Hu H, Chu M, et al. Revisiting the latent structure of negative symptoms in schizophrenia: evidence from two second-generation clinical assessments. Schizophr Res 2022; 248: 131–9. 36037646 10.1016/j.schres.2022.08.016

[5] Rucci P, Caporusso E, Sanmarchi F, Giordano GM, Mucci A, Giuliani L, et al. The structure stability of negative symptoms: longitudinal network analysis of the Brief Negative Symptom Scale in people with schizophrenia. BJPsych Open 2023; 9(5): e168. 37674282 10.1192/bjo.2023.541 PMC10594087

[6] Wolpe N, Chen S, Kirkpatrick B, Jones PB, Jenkins C, Cardinal RN, et al. Longitudinal effect of clozapine-associated sedation on motivation in schizophrenia: naturalistic longitudinal study. Br J Psychiatry 2023; 223: 295–7. 36624935 10.1192/bjp.2022.191 PMC10331318

